# Using the fsQCA approach to investigate factors affecting university students’ satisfaction with online learning during the COVID-19 pandemic: A case from China

**DOI:** 10.3389/fpsyg.2023.1123774

**Published:** 2023-02-27

**Authors:** Changhong Teng

**Affiliations:** Institute of Education, University College London, London, United Kingdom

**Keywords:** COVID-19, online learning, factors, students’ satisfaction, the fsQCA approach

## Abstract

During the COVID-19 pandemic, higher education institutions have been forced to switch their teaching mode to online education. There has been limited in-depth exploration of the factors affecting students’ satisfaction with online learning, and no consensus has been reached among these studies’ results. Students’ satisfaction is essential for realizing effective online education practices and meaningful to promoting the sustainable development of online courses, and it cannot be fully explained by one single factor. Research exploring the configuration of factors affecting students’ satisfaction with online learning has been rare. This study adopted a novel data analysis method, the fuzzy-set Qualitative Comparative Analysis (fsQCA) method, to explore collocations of different factors affecting higher education students’ satisfaction with online learning during the COVID-19 pandemic. This research surveyed 357 university students in Mainland China during the second semester of the 2021–2022 academic year using a structured questionnaire. The study identified that when students were satisfied with assignments and had a higher level of internet self-efficacy, or they were satisfied with their instructors and assignments, they were satisfied with online classes. Additionally, internet self-efficacy is indispensable to explaining students’ higher level of satisfaction with online learning. This study contributes to our understanding of university students’ satisfaction with online learning during the COVID-19 pandemic by using a novel method to explore the configuration of influential factors, and it provides implications for administrators and policymakers in the education field who seek to improve students’ satisfaction with online learning.

## 1. Introduction

### 1.1. Research background

At the end of 2019, Corona Virus Disease 2019 (COVID-19) appeared in China. As the virus spread, the World Health Organization (WHO) designated it a pandemic (WHO, 2020). Since then, the ongoing pandemic has been seriously disruptive to teaching/learning activities worldwide (Sharma et al., 2020), and nearly 90 percent of the world’s students have been influenced (UNESCO, 2020). Under this circumstance, school closures have been implemented at all levels from preschool to higher education (Simsek et al., 2021), and most higher education institutions around the world shut down their campuses in late March 2020 (Wong et al., 2020). As a result, higher education institutions have been forced to switch to an online teaching mode. Online learning did not originate during the COVID-19 pandemic, and higher education students had been exposed to this teaching mode for many years before the unprecedented closure. However, online learning on such a large scale is unprecedented in the history of higher education. Compared with normal online classes, this emergency remote teaching/learning could not be well-planned instructors do not have enough time. An important strand of the education literature has focused on online learning issues during the COVID-19 pandemic, especially issues related to students’ online learning.

Research has indicated that online learning has been a must for educational institutions during the pandemic to limit the disruption of education, as online learning is accessible, affordable, and flexible (Faize and Nawaz, 2020). Maqableh and Alia (2021) confirmed that safety and effectiveness are the two most important positive aspects of online learning. By taking an online approach, students and teachers have been less likely to catch the COVID-19 virus. However, the problems of online learning during this special period cannot be ignored. First, from a macro viewpoint, the transition to online learning during the pandemic has aggravated education inequalities. Online learning’s dependency on technological devices and the internet cannot be ignored. Accordingly, instructors and students with bad internet connections have been more likely to be excluded from online learning (Adedoyin and Soykan, 2020). Agormedah et al. (2020) also proposed that students’ ability to have access to e-learning devices and internet connectivity would determine the success of the students’ shift to remote learning. However, students in economic and financial distress are less likely to afford an unlimited and stable internet connection. This result buttressed the study of Adedoyin and Soykan (2020), which found that the online approach was more accessible for advantaged students with access to technology and hindered the participation of disadvantaged learners. Second, from a micro viewpoint, students with access to online learning also have faced challenges such as distraction, workload, technology issues, and inadequate support (Maqableh and Alia, 2021). Similarly, Faize and Nawaz (2020) also reported that students faced challenges such as a lack of resources and internet services and a lack of interaction. Because of these drawbacks to online learning, Mahyoob (2020) found that online learning had failed to allow students to fulfil their expected progress in learning performance, and that most learners were not satisfied with continuing online learning.

There is no denying that online practice is irreplaceable in circumstances such as the COVID-19 pandemic. Still, the problems that impede effective online education should be given more attention, especially those that decrease students’ satisfaction with online learning. In the short term, students’ satisfaction is really important for realizing effective online education practices (Sharma et al., 2020) and meaningful for promoting the sustainable development of online courses. As of May 2022, the epidemic had lasted for more than 2 years. It is uncertain how long the pandemic period will continue. Students have to prepare for long-term online learning as the epidemic becomes normalized. It is indispensable to investigate the factors influencing students’ satisfaction with online learning during the pandemic and to improve the negative aspects of this type of learning. In the long term, this situation will accelerate the transformation of traditional pedagogical approaches and force academic institutions to shift to online teaching and learning (Adedoyin and Soykan, 2020). One day, education activities may shift entirely to online teaching and learning, even if the epidemic is resolved. Students’ adoption of online education and their satisfaction with it is essential for the sustainable development of online learning. Therefore, exploring factors behind students’ dissatisfaction with online learning and dealing with these factors can further improve education equity in different regions.

### 1.2. Research gap

Few researchers have realized that some studies should be conducted to investigate students’ satisfaction with online learning during the pandemic. There has been limited in-depth exploration of the factors affecting students’ satisfaction with online learning, and no consensus has been reached among these studies’ results about students’ satisfaction. Furthermore, prior studies exploring the configuration of factors influencing students’ satisfaction with online learning during the COVID-19 pandemic in particular have been rare. In terms of research method, the existing studies have used fairly similar approaches; they have mostly been quantitative studies using methods such as cross-sectional design (Faize and Nawaz, 2020; Simsek et al., 2021) or structural equation models (Jiang et al., 2021; Kumar et al., 2021). Although some studies have adopted mixed methods (Landrum et al., 2021), it is challenging to find a single studying using a qualitative comparative analysis method. Motivated by these considerations, this research aimed to explore the factors affecting higher education students’ satisfaction with online learning during the COVID-19 pandemic using the fuzzy-set Qualitative Comparative Analysis (fsQCA) method. The basic idea of the fsQCA method is to analyze the influence of multiple antecedent variable combinations on result variables using Boolean operation. In recent years, the fsQCA method has gained increasing attention because it can explore the influence of collocations of different conditional variables on specific outcome variables. It focuses on the joint action of multiple influencing factors and provides a variety of combinations of condition variables. According to previous research, student satisfaction with online learning during the pandemic is influenced by multiple factors (Baber, 2020; Faize and Nawaz, 2020; Sharma et al., 2020; Jiang et al., 2021; Maqableh and Alia, 2021; Simsek et al., 2021). Student satisfaction may not be fully explained by individual factors acting alone, and the fsQCA method could explore collocations of different factors influencing student satisfaction. Therefore, this research utilized the fsQCA method to analyze student satisfaction using combinations of various factors.

Additionally, many types of research investigating student satisfaction have been conducted in Asian higher education settings. For example, Almusharraf and Khahro (2020) explored the satisfaction of 283 students from the Kingdom of Saudi Arabia with online learning. Besides, Simsek et al. (2021) investigated students’ satisfaction with online education in Turkey. Nevertheless, student satisfaction with online learning during the pandemic needs to get attention in other Asian countries, especially in countries such as China that have taken positive actions to combat the difficulties with online learning during the COVID-19 pandemic. To be specific, the Ministry of Education of the People’s Republic of China (2020a) launched an emergency policy called “Suspending Classes Without Stopping Learning” to ensure that students could continue to study at home through online learning platforms on January 29, 2020. From January 29 to April 3 of that year, 1,454 Chinese universities began their spring semester using online learning platforms. More than 950,000 university teachers offered more than 7,133,000 lectures on online learning platforms (Ministry of Education of the People’s Republic of China, 2020b). The Chinese government also took prompt action to guarantee the availability of fast and stable network services to students to make online education successful (Zhang et al., 2020). This research attempted to measure the factors affecting university students’ satisfaction with online learning during the COVID-19 pandemic in China using the fsQCA approach to provide implications for administrators and policymakers in the education field. This study also provides possible suggestions arising from the findings, especially for the future development of online learning.

### 1.3. Research questions

The research questions guiding this study were:

1. To what extent have Chinese higher education students been satisfied with online learning during the COVID-19 pandemic?
2. What factors have affected Chinese higher education students’ satisfaction with online learning during the pandemic?
3. How can these students’ satisfaction with online learning be improved?

## 2. Literature review

### 2.1. Concept of student satisfaction and influencing factors

Satisfaction is the pleasure or disappointment generated by comparing the perceived relationship Satisfaction is the pleasure or disappointment generated by comparing the perceived relationship between performance and expectations (Kotler and Keller, 2011). Student satisfaction can be defined as students’ perception of the university experience and the perceived value of education in educational institutions (Astin, 1993). As a short-term attitude, student satisfaction originates from a student’s evaluation of their own educational experience (Elliott and Healy, 2001). Considering all factors, Weerasinghe and Fernando (2017) concluded that student satisfaction refers to students’ short-term attitude based on their evaluation of their educational experience, services, and facilities.

Researchers have held different views when regarding the factors affecting higher education students’ satisfaction. Astin (1993) identified that contact time with teachers and administrators, career consultants, campus life, and relationships with teachers and administrators are important. However, Mai (2005) found that the most influential factors are the overall impression of the school, education quality, teachers’ expertise, the quality and availability of technical facilities, and the prospect of career development. Wilkins and Balakrishnan (2013) believed that lecturers’ quality, the quality of facilities, and the effective use of technology are important. Additionally, feedback quality, lecturer–student relationship, interaction between students, and availability of learning equipment and learning materials greatly affect student satisfaction (García-Aracil, 2009; Sojkin et al., 2012). It can be seen that students’ satisfaction is influenced by multidimensional factors and that influential factors in traditional face-to-face classes are relationships with faculty members, curriculum and instruction, campus life, support services, and facilities (Bolliger and Martindale, 2004). Compared with traditional classes, online courses bring challenges to instructors and students. Therefore, exploring student satisfaction within online settings is worthwhile because technologies have changed students’ interaction modes (Kaminski et al., 2009).

### 2.2. Review of previous literature on online learning students’ satisfaction during the COVID-19 pandemic

Various studies have provided useful insights into online learning students’ satisfaction during the COVID-19 pandemic (Baber, 2020; Faize and Nawaz, 2020; Sharma et al., 2020; Jiang et al., 2021; Maqableh and Alia, 2021; Simsek et al., 2021). Some studies have focused on students from one country and even from a single higher education institution. Sharma et al. (2020) explored online learning students’ satisfaction and its predictors among students at Chitwan Medical College. The results showed that more than half of the students were satisfied with online learning. Learners’ characteristics, technological characteristics, instructors’ characteristics, management, and coordination were factors significantly related to students’ satisfaction with online learning. These researchers also found that gender, Wi-Fi, and other network forms impact learners’ online learning satisfaction.

Regarding instructors’ characteristics, Sultana and Khan (2019) concluded that teachers’ performance significantly influences students’ satisfaction with online learning. Gender as an influencing factor has also been pointed out by Simsek et al. (2021), and they revealed that gender, discipline, education level, and grade level could cause changes in online learning students’ satisfaction levels during the pandemic. However, gender is a demographic feature that cannot be influenced or changed from the outside to improve the online learning process further. Furthermore, the other variables in Simsek et al.’s (2021) research—discipline, education level, and grade level—are also demographic features. Factors that are demographic features will not be considered in this research.

There have also been studies that have compared students from different backgrounds when exploring student satisfaction levels and the factors affecting them. For example, Jiang et al. (2021) explored online learning satisfaction in higher education by comparing students from two Chinese universities. The findings from this study showed students’ computer self-efficacy directly impacted their satisfaction with online learning. In addition, Baber (2020) conducted a cross-country study examining the determinants of undergraduate student satisfaction in South Korea and India. The study found that interaction, study motivation, course structure, instructor knowledge, and facilitation positively influence student satisfaction. Baber (2020) also found that there is no significant difference in student satisfaction between South Korea and India. Sharma et al.’s (2020) results supported instructor knowledge as a factor influencing student satisfaction. Faize and Nawaz (2020) also pointed out the importance of interaction to students’ satisfaction. They measured students’ satisfaction levels using Kranzow’s (2013) guidelines as a theoretical framework. Kranzow (2013) addressed students’ satisfaction through two channels. The first one was faculty–student interaction, which ensures the course content and the technology required to attend online courses are accessible to students. If students have any technical problems, the instructor will assist them in solving them. The second was the interaction between students and their peers. However, Maqableh and Alia (2021) revealed that reduced focus and psychological and administrative issues are also influential factors. This result is different from the findings of other previous literature reviewed in this section. In addition, there are many contradictions in their findings. For example, Maqableh and Alia (2021) found that more than a third of their participants were dissatisfied with their online learning experience. They identified that the respondents were not satisfied that they had more assignments. However, Simsek et al. (2021) found that students’ satisfaction level with online learning during the pandemic was moderate.

It can be deduced from the above-listed literature that previous studies have had inconsistent findings regarding the factors influencing student satisfaction with online learning during the COVID-19 pandemic, although there have been some overlapping factors. Besides, no study has considered combinations of various causal factors rather than single factors influencing student satisfaction.

### 2.3. Conceptual framework

This research explored how the non-demographic variables mentioned in section 2.2 (instructors, assignments, interactions, faculty services, and students’ internet self-efficacy) that have been proved to separately impact students’ satisfaction with online learning during the COVID-19 pandemic work as configurations. The conceptual framework for this research was based on Astin’s (1984) theory of student involvement.

Astin (1984) put forward his student involvement theory while investigating how the environment affects students’ learning and development. “Student involvement” refers to students’ physical and psychological effort in learning. A highly involved student will devote more time and energy to their studies, actively participate in various school activities, and interact with their classmates, instructors, and other staff. Accordingly, their satisfaction with school life will also improve. Astin’s theory has three key points: students’ input, environment, and output.

Students’ input refers to their potential and characteristics, family background, and academic and interpersonal relationships before the learning process. Environment refers to students’ actual experience in educational activities, including management strategies and practices in schools, courses, teaching, peer relations, and campus facilities. Output refers to educational activities’ direct and indirect impact, including students’ personality, knowledge, skills, attitudes, and behaviors. Astin’s theory emphasizes the role of the school environment, and a supportive environment is essential. In addition, it also pays attention to students’ non-classroom input. Although high-quality non-classroom experience is not an integral part of the curriculum, it can effectively complement education at school. In this research, students’ input factors were assignments and students’ internet self-efficacy. In addition, interaction and services provided by faculty were environmental factors. Students’ satisfaction with online learning during the COVID-19 pandemic was the result. Under this conceptual framework, the following sections will review instructors, assignments, interactions with instructors and other students, faculty services, and students’ internet self-efficacy.

#### 2.3.1. Instructors and student satisfaction

Eom et al. (2006) argued that instructor guidance and knowledge significantly affect student satisfaction. A study in Bangladesh showed that teachers’ performance is an essential factor affecting students’ satisfaction with online classes (Sultana and Khan, 2019). In a traditional setting, instructors and students can get immediate feedback on the course quality, delivery, and experience (Nambiar, 2020). Therefore, teachers can immediately adjust teaching methods by observing non-verbal cues from students to meet the students’ needs. However, instructors have to pay more attention and be more alert in an online class in order to notice what might be easily perceived in a face-to-face classroom. When it comes to the role of teachers in online learning, they have to encourage, guide, and stimulate students’ critical thinking rather than using traditional teaching methods (Huynh, 2005). Faize and Nawaz (2020) believed that instructors’ leadership is essential to students’ satisfaction during the online learning process. Meanwhile, apart from teaching techniques, Elshami et al. (2021) proposed that an excellent instructor in an online environment needs to have stable technical equipment. Thus, as a factor affecting student satisfaction with online courses, instructors have to face the challenges of online learning and upgrade their teaching and technical skills.

#### 2.3.2. Assignment and student satisfaction

Online assessment positively affects student satisfaction with online learning (George, 2020). There are various techniques for online assessment, like online tests, essays, presentations, quizzes, assignments, etc. However, assignments were the most used assessment method during the COVID-19 pandemic (Ebohon et al., 2021; Şenel and Şenel, 2021). Therefore, assignments, as the most popular assessment method, affect students’ satisfaction with online learning. Many students pointed out the problems of assignments during the pandemic, such as the workload, instructor feedback, ease of submitting/responding, etc. Students from a university in Abu Dhabi in the United Arab Emirates considered a heavy amount of coursework a negative aspect of their online learning (Hussein et al., 2020). This was supported by the research of Maqableh and Alia (2021), who found that 82.5% of their respondents reported that they had more assignments and that the classwork became homework. Besides, Aristovnik et al. (2020) surveyed 30,383 students in 62 countries about their perception of their coursework. Nearly half (42.6%) of these students reported their workload during the pandemic had increased compared to before. Regarding feedback, Eom and Ashill (2016) concluded that instructor feedback to students could enhance student satisfaction by improving learner affective responses and increasing their cognitive skills. Students need more interaction and feedback when completing assignments, and they also demand well-defined instructions (Şenel and Şenel, 2021). Thus, assignments are a really important factor to investigate when researching university students’ satisfaction with online learning during the COVID-19 pandemic.

#### 2.3.3. Interaction and student satisfaction

In online settings, students may never access physical campus locations and may have difficulty building relationships with faculty and classmates (Bolliger and Martindale, 2004). New technologies have also changed how students interact with teachers and classmates (Kaminski et al., 2009). As for the influence of interaction, Moore (2002) found that the interaction between instructors and learners was the most important factor in student satisfaction. Besides, Vonderwell and Turner (2005) also identified that positive teacher–student interaction is a factor that affects satisfaction levels. Wu et al. (2010) found student and instructor interaction to be significantly positively related to the online learning climate and performance expectations, which in turn contributed to student satisfaction. Both Li et al. (2016) and Price et al. (2016) found interactions (between student and teacher) were positively related to student satisfaction. According to most online learning studies, learner–learner interaction and learner–instructor interaction seem related to student satisfaction (Jung et al., 2002; Bolliger and Martindale, 2004). Therefore, interaction in this research included learner–lecturer and learner–learner interaction.

#### 2.3.4. Faculty services and student satisfaction

Moore and Kearsley (1996) pointed out that administrative support is very helpful to distance learning students. They suggested that students should have a contact person who can help them. This may be because students without technical support may experience severe frustration in an online environment (Hara, 2000). Beyond the importance of students interacting with the teacher, the role of other faculty has also been shown to impact student satisfaction. Most students were satisfied with the prompt response from the concerned faculty and departments about internet issues. Therefore, faculty support, as a factor affecting student satisfaction with online courses, is indispensable.

#### 2.3.5. Internet self-efficacy and student satisfaction

Internet self-efficacy is the belief that one can organize and perform internet-related actions required to complete specified tasks (Eastin and LaRose, 2000). Students have to use reliable equipment and be familiar with the techniques used in their courses to achieve success in online courses (Belanger and Jordan, 1999). Research has shown that students with higher levels of internet self-efficacy have better information search skills (Thompson et al., 2002), which may enhance their confidence in using the internet and solving problems in the learning process. Internet self-efficacy is a critical factor in student satisfaction. Students who are up to date with the appropriate technologies can continue their studies smoothly and with greater satisfaction during online learning (Johnson et al., 2016). In contrast, students who experienced frustration with technology reported lower satisfaction levels (Hara, 2000).

### 2.4. Summary and research direction

This section reviewed a representative sample of the available literature about student satisfaction and, in particular, student satisfaction with online learning during the COVID-19 pandemic. It can be concluded from the literature that various factors can influence student satisfaction, not only in traditional teaching modes but also in an online setting. Various studies on student satisfaction with online learning have investigated the following factors: instructor interaction, communication, students’ ability to control their actions in the learning environment, efficient assessment, and technology (Cole et al., 2014; Dziuban et al., 2015). However, the research thus far has explored student satisfaction during the COVID-19 pandemic on the surface without in-depth analysis. Student satisfaction with online learning during the COVID-19 pandemic may not have been fully explained. Therefore, this research explored the combinations of various causal factors (instructors, assignments, interaction, faculty services, and internet self-efficacy).

## 3. Materials and methods

This research aimed to explore to what extent Chinese higher education students have been satisfied with online learning during the COVID-19 pandemic and what factors have affected their satisfaction levels. We chose a qualitative method to investigate the factors affecting higher education students’ satisfaction with online learning during the COVID-19 pandemic because qualitative methods are widely used in education research (Divan et al., 2017). This study employed a survey that was administered to a group of Chinese students. The survey mainly consisted of a set of 5-point Likert scale questions concerning the students’ online experience. I employ self-administered questionnaires to collect empirical data. Moreover, Maguire and Delahunt (2017) claimed that data analysis is the core of the credibility of qualitative research and statistical analyses should be based on the type of data collected and the goals of the survey. The current research investigated the configuration of factors influencing students’ satisfaction with online learning. In order to better suit our purpose, we adopted the fsQCA method to analyze our data. In the following subsections, we will describe: (1) the research design, (2) data collection, (3) instrument, and (4) measurements.

### 3.1. Research design

**Figure 1:**
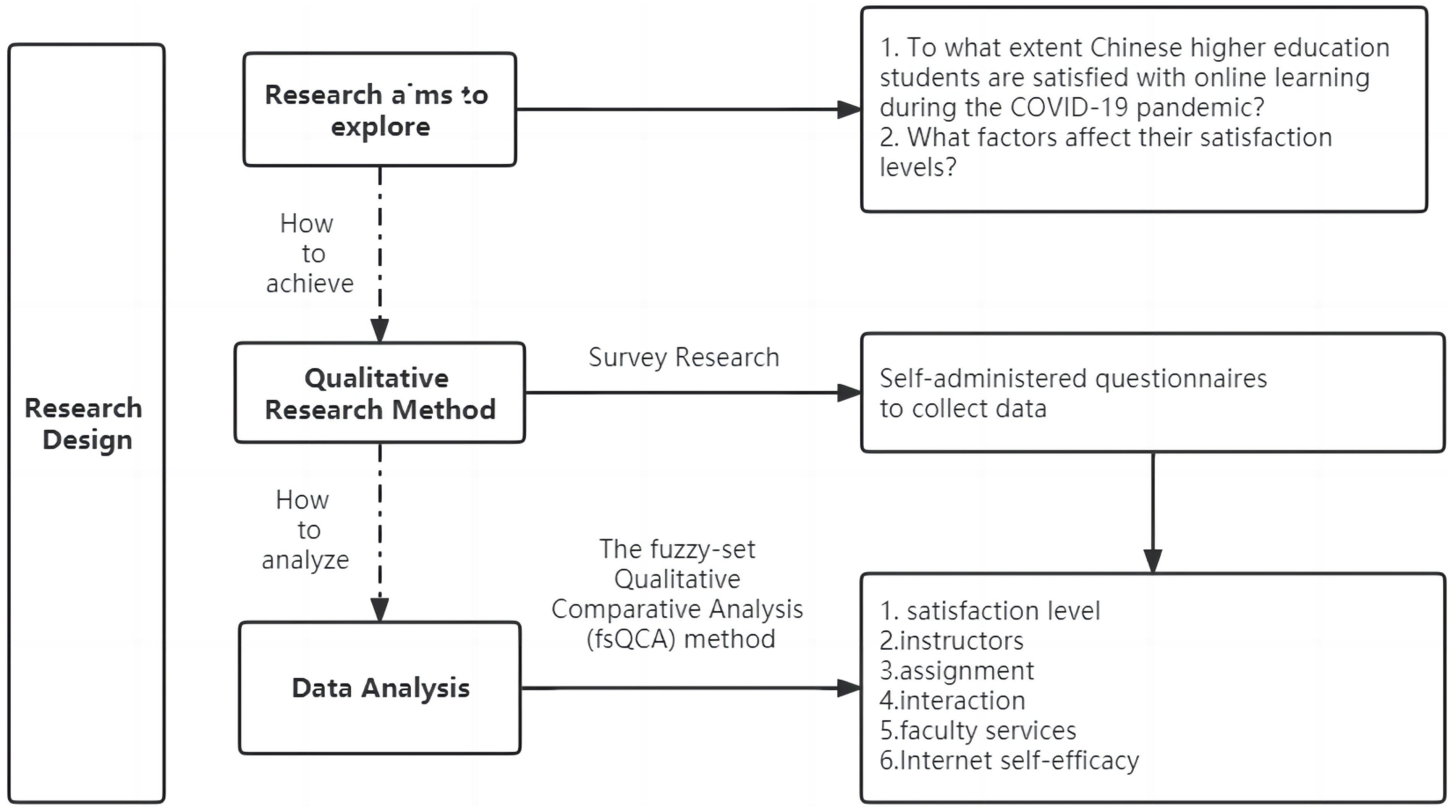
Research design.

In order to obtain empirical data for this study, self-administered questionnaires were distributed to Chinese higher education students. We believe that this survey research approach was suitable for the data we wanted to collect and could answer the research questions mainly due the following considerations. First, a questionnaire was easy to implement, and it allowed us to collect data online during the pandemic. Also, the results of the questionnaire were convenient for statistical processing and analysis. Figure 1 shows an image of the research design.

When it came to data analysis, we adopted the fsQCA method, a set-theoretical approach introduced by Ragin (2000) and recently applied increasingly in many fields. The method is intended to analyze the influence of multiple antecedent variable combinations on result variables using Boolean operations. Additionally, it focuses on the joint action of multiple influencing factors and provides a variety of combinations of condition variables. Unlike other correlation-based quantitative methods, the fsQCA method seeks to establish a logical link between combinations of causal conditions (conjunctural causation) and an outcome, thereby generating rules that summarize the adequacy of all possible combinations of subsets based on causal conditions (or their supplements) and the outcome (Mendel and Korjani, 2012). The fsQCA approach attempts to avoid some limitations of multiple regression. Woodside (2013) proposed that a negative or positive relationship can exist between the two variables. Still, regression analysis does not account for or explain all cases in a dataset, as in any given dataset, not all cases support a negative or positive relationship between the independent and dependent variables (Skarmeas et al., 2014).

In order to overcome the defects of existing research on students’ satisfaction, this study adopted the fsQCA method to analyze this construct. Kraus et al. (2018) argued that fsQCA is a method that employs qualitative inquiry with quantitative exploration to explicate complex phenomena by using configuration analysis. It can be concluded from the literature review that various factors can influence student satisfaction with online learning during the pandemic (instructors, assignments, interaction, faculty services, and internet self-efficacy). The fsQCA approach in this research could identify all necessary and sufficient conditions that lead to student satisfaction with online learning.

### 3.2. Data collection and research sample

This research utilized a survey administered to university students from 10 colleges and universities in Mainland China during the second semester of the 2021–2022 academic year. In May 2022, the anonymous questionnaire was distributed *via* an online survey system (Wenjuanxing). The snowball method of sampling was used for recruitment: We gave each participant a discount coupon when they shared the questionnaire link with their classmates—the coupon was an incentive. Snowball sampling is suitable for sampling members of a special population segment. In our research, we needed to collect data from Chinese university students. It was really hard for us to get enough data through sending questionnaires to students because of the school closures during the pandemic. In such a case, snowball sampling was the best sampling method for us to choose.

In total, 424 questionnaires were collected, of which 67 were invalid (including 49 samples in which the same option was chosen continuously, five in which the responses were logically contradictory, 10 samples obtained from postgraduates, and three that were self-test samples); finally, there were 357 valid samples. Table 1 shows the demographic characteristics of the valid samples. As one can see, 48.7% (174) of participants were male, and 51.3% (183) were female. As the participants were higher education students, the age of almost 71.4% (255) of them was between 19 and 24 years old, 18.8% (67) of them were under 18 years old, and about 9.8% (35) of them were between 25 and 30 years old. At the same time, all participants were undergraduate students. Regarding the residential area where these students lived, the number (148) of those who lived in an urban area was nearly the same as that of those from a smaller town (142). Additionally, 18.8% (67) of these respondents were from rural areas.

**Table 1:** Demographic characteristics of students.

Characteristics	Frequency	Percentage (%)
Gender		
Male	174	48.7%
Female	183	51.3%
Age of students		
Under 18	67	18.8%
19–24 years	255	71.4%
25–30 years	35	9.8%
Level of study		
Freshman	93	26.1%
Sophomore	88	24.6%
Junior	106	29.7%
Senior	70	19.6%
Residential area		
Urban	148	41.4%
Town	142	39.8%
Rural	67	18.8%

### 3.3. Instrument

The questionnaire consisted of four domains: demographic information, academic background, perceptions of online learning from the learners’ perspectives, and satisfaction with online learning. The first 11 questions included in the first part of the survey were intended for understanding the respondents’ demographic information. Then, there were multiple-choice questions for learning about participants’ academic background (seven items). In the third part, six questions revealed whether the students were able to participate in face-to-face classes that semester and their difficulties with online learning. Finally, six questions were used to identify their satisfaction with online learning. Each item in the last domain was rated on a 5-point Likert scale (1: *strongly disagree*, 2: *disagree*, 3: *neutral*, 4: *agree*, 5: *strongly agree*). Before issuing the questionnaire, we invited three higher education students from different disciplines (who were not included in the final analysis) to pretest the questionnaire.

#### 3.3.1. Variable selection

Configuration analysis mainly studies how variables work together to influence the result. Variable selection is really important.

The existing methods of constructing configurations are the inducive method and the deductive method. The inducive method requires researchers to judge and extract relevant conditional variables based on existing knowledge and research conclusions. In contrast, the deductive method is based on a theoretical framework or theory containing configuration conditions. As described in the conceptual framework in 2.3, Astin’s (1984) theory emphasizes that highly involved students tend to be more satisfied with school life. Under the conceptual framework, instructors, assignments, interaction, faculty services, and students’ internet self-efficacy were condition variables in this research. Instructor guidance, knowledge, and performance significantly affect student satisfaction. During the pandemic, instructors have had to upgrade their teaching and technical skills to adopt the online teaching mode. The instructor as a condition variable was abbreviated as SS. Regarding assignments, the workload, ease of upload, and feedback on assignments were really important to the investigation of university students’ satisfaction with online learning during the pandemic. Assignments were abbreviated as AS in this research. As for interaction, learner–learner interaction and learner–instructor interaction are predictive of student satisfaction. IT was the abbreviation for interaction in this research. Likewise, faculty services are helpful to distance learning students and influence their satisfaction. Internet self-efficacy is also a critical factor in student satisfaction. Students with higher internet self-efficacy tend to be more satisfied with online learning. FS and IS were the abbreviations for faculty services and internet self-efficacy in this study. As discussed above, we used the fsQCA approach to investigate these factors affecting university students’ satisfaction with online learning during the COVID-19 pandemic. Therefore, the outcome variable was student satisfaction with online learning during the pandemic. The condition and outcome variables and their abbreviations are listed in Table 2.

**Table 2:** Condition variable and outcome variable.

Type	Name	Abbreviation
Outcome variable	Student Satisfaction	SS
Condition variable	Instructor	IN
	Assignment	AS
	Interaction	IT
	Faculty services	FS
	Internet self-efficacy	IS

#### 3.3.2. Pre-processing

To meet the Boolean logic of qualitative comparative analysis, it was necessary to calibrate the data according to relevant standards before the analysis to make the results interpretable. Therefore, it was essential to transform the variables’ values from the numerical sample data from the 5-point Likert scale to fuzzy-membership scores over the consistency between 0 and 1 (Woodside et al., 2011). This study solved this problem using the direct method (see Ragin, 2009) and drew on researcher-specified threshold qualitative anchors to determine full membership (Maximum Threshold), the crossover point, and full non-membership (Minimum Threshold). Evaluation of the three qualitative anchors for each variable came from Andrews et al. (2016) and involved the identification of the 5th percentile (Minimum Threshold), 95th percentile (Maximum Threshold), and 50th percentile (crossover point) values. First, the average of each item was taken as the initial data for each variable. Secondly, three anchor points needed to be identified: the full membership value (1), crossover point (0.5), and full non-membership value (0). The membership degree of the calibrated set was between 0 and 1. The calibration anchor points of each variable are shown in the following Table 3. The minimum threshold, crossover point, and maximum threshold of student satisfaction were 1.8, 4.0, and 4.6, respectively. The minimum threshold and the crossover point of the five condition variables (Instructors, Assignment, Interaction, Faculty services, and internet self-efficacy) were the same (2.2 and 4.0). The maximum threshold of these condition variables were 4.6, 4.7, 4.5 4.6, and 4.6, respectively.

**Table 3:** Description of anchor point threshold of FuzAS set.

Variable	Minimum threshold	Crossover point	Maximum threshold
Student satisfaction (SS)	1.8	4.0	4.6
Instructors (IN)	2.2	4.0	4.6
Assignment (AS)	2.2	4.0	4.7
Interaction (IT)	2.2	4.0	4.5
Faculty services (FS)	2.2	4.0	4.6
Internet self-efficacy (IS)	2.2	4.0	4.6

This research used fsQCA3.0 software to analyze the configurations of factors within 357 samples affecting higher education students’ satisfaction with online learning during the COVID-19 pandemic in China. The operation of fsQCA include three steps:

- The necessity analysis of single-condition variables
- The construction of the truth table
- Configuration analysis

The outcomes of the fuzzy-set analysis were represented by special software symbols. Specifically, black circles (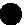) meant the presence of a condition, while crossed-out circles (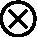) denoted its absence (Fiss, 2011). Large circles marked the core elements of a configuration, and the peripheral elements were marked with small circles. In addition, blank spaces indicated a do-not-care situation in which the causal condition may be either present or absent. Consistency measured the degree to which each condition variable was a subset of the outcome variable, and coverage measured the empirical relevance of a consistent subset (Ragin, 2006; Mendel and Korjani, 2012).

### 3.4. Measurements

In this study, the variables were measured using a 5-point Likert scale, and each variable was measured using the average score of the corresponding items on the scale. SPSS 25.0 was used to analyze the reliability and validity of the scale items. Table 4 shows the reliability and validity of the scale. First, the Cronbach’s coefficients of all six scales were greater than 0.8, indicating that the scale questionnaire had good reliability. Secondly, the KMO values of these scales were more than 0.8, and the cumulative variance contribution rates were more than 60%, indicating that the scale questionnaire had good content validity. In general, the factor loading of each item was above 0.5, the combined reliability (CR) of each item was greater than 0.6, and the average extraction variance (AVE) was greater than 0.5, indicating that the questionnaire had good convergent validity. In conclusion, the scale had good reliability and validity and could be used for further research.

**Table 4:** Reliability and validity analysis table.

Variable	Item	Factor loading	Cronbach’s	KMO	CR	Cumulative variance (%)	AVE
Student satisfaction	1	0.829	0.901	0.881	0.901	71.672	0.646
	2	0.77					
	3	0.793					
	4	0.799					
	5	0.826					
Instructors	1	0.745	0.881	0.81	0.886	68.649	0.608
	2	0.791					
	3	0.791					
	4	0.793					
	5	0.778					
Assignment	1	0.797	0.916	0.916	0.914	70.046	0.641
	2	0.806					
	3	0.809					
	4	0.774					
	5	0.81					
	6	0.763					
Interaction	1	0.793	0.901	0.898	0.901	66.916	0.603
	2	0.762					
	3	0.797					
	4	0.763					
	5	0.82					
	6	0.733					
Faculty services	1	0.805	0.893	0.874	0.893	70.081	0.627
	2	0.79					
	3	0.808					
	4	0.788					
	5	0.776					
Internet self-efficacy	1	0.793	0.894	0.877	0.895	70.371	0.63
	2	0.797					
	3	0.813					
	4	0.805					
	5	0.78					

## 4. Results and findings

### 4.1. To what extent are Chinese higher education students satisfied with online learning during the pandemic?

According to Table 5, 31.4% of these students preferred online learning to the face-to-face learning mode, and 23.2% strongly agreed that they preferred online learning. As for the continuation of online learning, 33.3% of the respondents agreed with it, and 21.9% strongly agreed. It can be inferred from the data that more than half of these respondents held a positive attitude toward the continuation of online learning in the future. This calls on universities to pay attention to student satisfaction with online learning and improve online learning from all aspects gradually.

**Table 5:** Whether Chinese higher education students were satisfied with online learning during the COVID-19 pandemic.

Item	Strongly disagree	Disagree	Neutral	Agree	Strongly agree	Average	Standard deviation
You are satisfied with online learning during the pandemic.	19 (5.3%)	47 (13.2%)	65 (18.2%)	130 (36.4%)	96 (26.9%)	3.66	1.16
You prefer the online learning mode to the face-to-face mode.	22 (6.2%)	43 (12.0%)	97 (27.2%)	112 (31.4%)	83 (23.2%)	3.54	1.15
You believe that online learning is more efficient.	18 (5.0%)	44 (12.3%)	74 (20.7%)	127 (35.6%)	94 (26.4%)	3.66	1.14
Online learning is more helpful to improve your academic performance.	21 (5.9%)	44 (12.3%)	114 (31.9%)	107 (30.0%)	71 (19.9%)	3.46	1.12
You hope to continue online learning mode in the future.	28 (7.8%)	46 (12.9%)	86 (24.1%)	119 (33.3%)	78 (21.9%)	3.48	1.19

**Figure 2:**
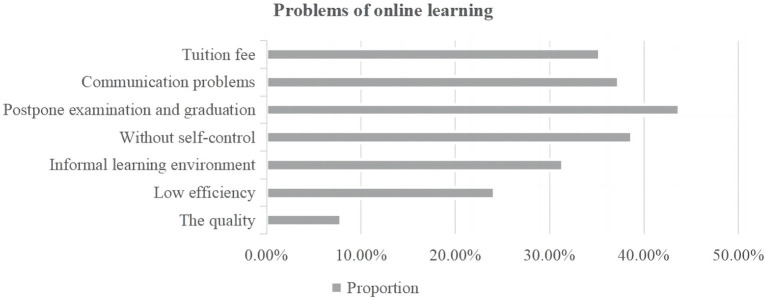
Concerns of online learning.

Table 5 shows the extent to which Chinese higher education students were satisfied with online learning during the pandemic. Each item was rated using a 5-point Likert scale (1: *strongly disagree*, 2: *disagree*, 3: *neutral*, 4: *agree*, 5: *strongly agree*). As can be seen from the table, the average point of students’ satisfaction with online learning was 3.66, which is between *neutral* and *agree*. It can be inferred from the data that many participants were not satisfied with online learning during the pandemic. However, nearly 36.4% of the respondents reported that they were satisfied with online learning, and 26.9% strongly agreed with that statement. In order to know the reasons why the remaining Chinese higher education students were not satisfied with online learning, our questionnaire included a multiple-choice question that asked respondents to identify the problems they faced during the online process. In Figure 2, one can see that deferred examination and graduation were what students cared about most (43.7%). This is a reminder that universities need to arrange examinations and graduation reasonably.

Additionally, universities should give students clear instructions and explain planning about examinations and graduation to avoid students’ concerns. Lower self-control ability and interaction with students and teachers were the second and third problems that students were concerned about (38.7 and 37.2%, respectively). Surprisingly, 35.3% of the respondents believed that the value for money of online courses was not high because the tuition for them was the same as for face-to-face courses. Students were also troubled by the informal learning environment and low learning efficiency (31.4 and 24.1%). These two problems can be solved effectively through students’ efforts, such as by finding a comfortable place to learn and planning their tasks to avoid procrastination and distraction. However, quality of online classes was the problem that students cared about least (7.8%). These results show that not only should universities provide technical support to students, but also teachers should improve the classroom atmosphere to help students concentrate.

### 4.2. The necessity analysis of single condition variable

Unlike the traditional regression method, fsQCA does not pay attention to the influence of a single variable on the results but focuses on the necessary conditions or sufficient condition combinations that lead to the outcome variables. As mentioned in 3.3, the operation of fsQCA includes three steps. After the above calibration, we used fsQCA to perform the necessity test of single-condition variables, and the results are shown in the Table 6. The analysis results of necessity are reflected in consistency and coverage. Consistency measures the degree to which each condition variable is a subset of the outcome variable. Coverage measures the coverage of each condition variable to the outcome variable degree. Both values are between 0 and 1. Generally speaking, when the consistency is greater than 0.9, this condition variable can be considered a necessary condition for the results.

**Table 6:** Necessity test of single condition variable.

Condition Variable	Consistency	Coverage
IN	0.762077	0.786288
~IN	0.593070	0.602955
AS	0.761752	0.775622
~AS	0.579092	0.596574
IT	0.787629	0.726426
~IT	0.527079	0.606842
FS	0.775688	0.716961
~FS	0.534550	0.613788
IS	0.752706	0.759129
~IS	0.578023	0.601309

“~” Represents “negation.”

It can be seen that the consistency of Instructors (IN), Assignment (AS), Interaction (IT), Faculty services (FS), and internet self-efficacy (IS) were all less than 0.9, which was not enough for any of them to constitute a necessary condition that affected Chinese university students’ satisfaction with online learning during the pandemic. This indicates that these variables would weakly explain the outcome variables independently. Therefore, it was necessary to analyze further the configurations of these condition variables and their impact on the outcome variables.

### 4.3. Configuration analysis results

This research used fsQCA3.0 software to analyze the configurations with 357 samples who responded about higher education students’ satisfaction with online learning during the COVID-19 pandemic in China. The consistency threshold, PRI consistency, and case threshold were set to 0.8, 0.876, and 9, respectively. Finally, we obtained three solutions: a complex solution, a parsimonious solution, and an intermediate solution. In addition to the differences in complexity, the three solutions were also different in terms of their enlightenment and universality. To be specific, the complex solution was the most comprehensive, but its universality was poor, resulting in a lack of applicability and generalizability of the conclusion. The parsimonious solution was the simplest one. Still, it lost more information in the process of omission, so the enlightenment of the conclusion was weak and might have been contrary to the actual situation. The explanatory power of the intermediate solution was between the former two. Generally, it represented the combination of the theoretical basis of the research with the analysis results. Therefore, it had strong enlightenment and universality. In general, the intermediate solution determines the final configuration result. Additionally, the intermediate solution and the parsimonious solution jointly determine the core conditions and peripheral conditions in the configuration. Fiss defined the conditions that appear in both a parsimonious solution and an intermediate solution as the core conditions and the conditions that appear in the intermediate solution but are excluded from the parsimonious solution as the secondary conditions.

In the complex solution, we got five path solutions. The solution coverage and solution consistency were 0.685704 and 0.844349, respectively. As for the parsimonious solution, there were three path solutions, and the solution coverage and solution consistency were 0.741548 and 0.802272, respectively. In addition, there were five paths in the intermediate solution. The complex solution, parsimonious solution, and intermediate solution of this research are listed in Table 7.

**Table 7:** Complex solution, parsimonious solution, and intermediate solution.

Item	Path of solutions	Raw coverage	Unique coverage	Consistency
Complex solution	IN*AS*IT*IS	0.51365	0.01826	0.897513
	IN*AS*FS*IS	0.48352	0.01886	0.890411
	AS*IT*FS*IS	0.50778	0.047	0.872046
	IN*AS* ~ IT* ~ FS* ~ IS	0.29764	0.04305	0.888975
	~IN* ~ AS*IT* ~ FS*IS	0.2887	0.035	0.885292
Solution coverage: 0.685704				
Solution consistency: 0.844349				
Parsimonious Solution	~FS*IS	0.41227	0.05903	0.814841
	AS*IS	0.62817	0.27492	0.846636
	IN*AS* ~ FS	0.3851	0.05435	0.870203
Solution coverage: 0.741548				
Solution consistency: 0.802272				
Intermediate Solution	IN*AS*IT*IS	0.51365	0.01826	0.89751
	IN*AS*FS*IS	0.48352	0.01886	0.89041
	AS*IT*FS*IS	0.50778	0.047	0.87205
	IN*AS* ~ IT* ~ FS* ~ IS	0.29764	0.04305	0.88898
	~IN* ~ AS*IT* ~ FS*IS	0.2887	0.035	0.88529
Solution coverage: 0.685704				
Solution consistency: 0.844349				

“*” Represents “plus.”

### 4.4. Results and analysis

Outcomes of the fuzzy-set analysis for students’ satisfaction with online learning during the pandemic are presented in Table 7. There were five configurations with which Chinese higher education students could achieve satisfaction with online learning during the COVID-19 pandemic. The overall consistency of these five configurations was higher than 0.8 (0.844349), which indicated that these five configurations fully explained satisfaction with online learning during the pandemic. The coverage of the solution was 0.685704, which means that these five configurations accounted for nearly 69% of the satisfaction with online learning during the pandemic among Chinese higher education students. Table 8 presents each configuration that constituted high satisfaction with online learning during the pandemic in fsQCA. Each configuration will be explained and discussed in detail in the following parts.

**Table 8:** Configurations with high satisfaction in fsQCA.

Condition variable	Satisfied with online learning during COVID-19 pandemic				
	Solution 1	Solution 2	Solution 3	Solution 4	Solution 5
IN	●	●		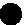	⊗
AS	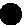	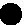	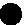	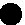	⊗
IT	●		●	⊗	●
FS		●	●	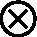	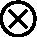
IS	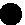	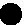	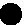	⊗	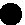
Raw coverage	0.513654	0.483522	0.507775	0.297642	0.288697
Unique coverage	0.0182617	0.0188579	0.0469952	0.0430468	0.0350017
Consistency	0.897513	0.890411	0.872046	0.888975	0.885292
Solution coverage: 0.685704					
Solution consistency: 0.844349					

#### 4.4.1. Assignment plus internet self-efficacy

Solutions 1–3 reflected combinations of the presence of assignment with internet self-efficacy, which allowed higher students’ satisfaction levels with online learning during the COVID-19 pandemic to occur. To be specific, Solution 1 showed that students’ satisfaction with assignments and higher internet self-efficacy, combined with relative satisfaction with the instructor and interaction, would lead to university students’ satisfaction with online learning, regardless of their level of satisfaction with faculty service. Likewise, the combination of satisfaction with assignments and higher internet self-efficacy, with relative satisfaction with the instructor and faculty service, would lead to university students’ satisfaction with online learning, regardless of their level of satisfaction with interaction (Solution 2). Solution 3 showed that, with the combination of the presence of assignments and internet self-efficacy, interaction and faculty services were peripheral conditions, and students would be satisfied with online learning regardless of their satisfaction level with the instructor.

Assignments and internet self-efficacy were the strongest factors affecting university students’ satisfaction with online learning during the COVID-19 pandemic, which confirms the findings of Jiang et al. (2021), Maqableh and Alia (2021), and Şenel and Şenel (2021). In terms of assignments as an influencing factor, on the one hand, the workload of assignments significantly affected students’ satisfaction with online learning. On the other hand, feedback on assignments was also important. Maqableh and Alia (2021) found that most students in their research were not satisfied with their increased number of assignments compared with traditional learning. There are multiple reasons for this increased number of assignments. During the COVID-19 pandemic, assignments became an important assessment method, which led students to have more assignments, and classwork became homework. Besides, teachers get limited feedback from students during online classes. Therefore, during online learning, assignments become one of the important means for teachers to understand students’ learning process. Teachers therefore request students to complete homework on more complex subjects, which puts extra pressure on the students. Additionally, students need more interaction and feedback when completing assignments. The influence of internet self-efficacy on student satisfaction with online learning during the COVID-19 pandemic was supported in the present study. However, there has been little research investigating internet self-efficacy and student satisfaction with online learning during COVID-19. Jiang et al. (2021) concluded that computer self-efficacy directly impacted Chinese university students’ satisfaction with online learning platforms. Kuo et al. (2013) also proposed that internet-self-efficacy was a good predictor of student satisfaction with online education. This study’s result, however, was contrary to that of Robles (2006), who found that internet self-efficacy was not an important factor in student satisfaction.

#### 4.4.2. Instructor plus assignment

In Solution 4, the combination of instructors with assignments in the absence of faculty service led to university students’ high satisfaction levels in online learning. Interaction and internet self-efficacy in Solution 4 were peripheral elements.

As for the instructor as a core factor in the present study, this result is in line with Fatani’s survey (Fatani, 2020). This survey investigated student satisfaction with online courses during the pandemic and pointed out that instructors’ teaching quality positively impacted student satisfaction. Baber (2020) also found that instructor knowledge positively influences student satisfaction, supporting Sharma et al. (2020). Sharma et al. (2020) explored satisfaction with online learning and its predictors among students. They concluded that instructors’ characteristics, management, and coordination by faculty are significantly related to student satisfaction. Landrum et al. (2021) also pointed out that the faculty’s role impacted student satisfaction beyond the teacher’s interaction. However, as for faculty service, contrary to the study of Sharma et al. (2020) and Landrum et al. (2021), this study found that even lower satisfaction levels of faculty services could lead to student satisfaction with online learning. The low estimated value of faculty service in this research might be due to the following reason: Students who have never met problems and have never needed to be assisted might think that the role of faculty is not significant to them since they have had to operate the learning system independently online. This result therefore requires further research to verify whether faculty service is a factor affecting university students’ satisfaction with online learning during the pandemic.

Assignment as an important factor has been discussed in 4.4.1. The following part of this section will discuss interaction as a peripheral factor in Solution 4. The result showed that interaction was not a critical factor, and relative satisfaction with interaction would lead to university students’ satisfaction with online learning. This result is inconsistent with that of Faize and Nawaz (2020), who pointed out the importance of interaction in student satisfaction with online learning. However, this result is in line with that of Landrum et al. (2021). They concluded that the amount of interaction with faculty and students is unimportant, but what is important is whether this interaction is expected and fits with the online experience. This differs from previous research about satisfaction with online learning before the pandemic. For example, Vonderwell and Turner (2005) identified active student–teacher interactions as affecting satisfaction levels.

#### 4.4.3. Internet self-efficacy subtract faculty services

In Solution 5, the absence of faculty services and the presence of internet self-efficacy were core elements determining students’ satisfaction levels in online learning during the COVID-19 pandemic. At the same time, instructor, assignment, and interaction were peripheral elements.

The absence of faculty services has been discussed in 4.4.2, and internet self-efficacy as an influencing factor has been discussed in 4.4.1. Internet self-efficacy was a core factor in Solution 5 that determined Chinese university students’ satisfaction with online learning during the pandemic.

## 5. Discussion

Due to the COVID-19 pandemic, higher education institutions around the world have faced near-total closures. Most educational institutions worldwide have offered online courses in the past 2 years. Under the ongoing pandemic, China has adopted a strict segregation policy to manage higher education institutions. Therefore, face-to-face education has been replaced by an online mode in most Chinese universities. Higher education institutions regard student satisfaction as one of the main factors determining the quality of online courses (Yukselturk and Yildirim, 2008). Students’ satisfaction is really important to the sustainable development of online learning in the future.

This research was an effort to investigate the configuration of factors affecting university students’ satisfaction with online learning during the COVID-19 pandemic. Current research has focused on whether any single factor has affected university students’ satisfaction with online learning during the COVID-19 pandemic using quantitative methods such as regression analysis or qualitative methods (Baber, 2020; Faize and Nawaz, 2020; Sharma et al., 2020; Jiang et al., 2021; Maqableh and Alia, 2021; Simsek et al., 2021). However, no previous study has performed an in-depth exploration of the configuration of factors affecting students’ satisfaction with online learning during COVID-19. This research adopted the fsQCA method to analyze the data collected from 357 university students in Mainland China during the second semester of the 2021–2022 academic year.

The research findings showed that the average degree to which students were satisfied with learning during the pandemic was 3.64, which was between *neutral* and *agree*. It can be inferred from the data that the participants were not, overall, satisfied with online learning during the pandemic. This result is different from those in previous literature. For example, Sharma et al. (2020) found that more than half of students were satisfied with online learning. Furthermore, Simsek et al. (2021) identified that students’ satisfaction level with online learning during the pandemic was moderate. Further, this study identified five configurations that led to a higher level of university students’ satisfaction with online learning during the COVID-19 pandemic. Solutions 1–3 showed that assignments combined with internet self-efficacy would lead to satisfaction with online learning. Previous studies only proposed that students had a heavy number of assignments during the pandemic (Aristovnik et al., 2020; Hussein et al., 2020), but they did not explore the influence of assignments on students’ satisfaction. In addition, there has been little research investigating internet self-efficacy and student satisfaction with online learning during COVID-19. Jiang et al. (2021) proposed that computer self-efficacy directly impacted Chinese students’ satisfaction with online learning platforms rather than with online learning itself. Compared with previous research, this current study revealed that students are satisfied with online learning when they are satisfied with assignments and internet self-efficacy at the same time. In Solution 4, the combination of the instructor with assignments was the core factor. This finding buttresses the study of Sultana and Khan (2019), who found that teachers’ performance is an essential factor affecting students’ satisfaction with online classes. However, Faize and Nawaz (2020) believed that instructors’ leadership, but not instructors themselves, has been essential to students’ satisfaction during the online learning process. Compared with previous research, this current study revealed that students are satisfied with online learning when they are satisfied with instructors and assignments at the same time. In addition, internet self-efficacy was indispensable in Solution 5 to explain the higher level of satisfaction with online learning. However, other factors that were the most influential factors on student satisfaction in previous research, such as interaction and faculty service, were not important in this research.

In order to answer the third research question (How can higher education students’ satisfaction with online learning be improved?), we received inspiration from the research findings. For the instructors, online teaching is a complicated transition process. Wasserman et al. (2020) argued that even good teachers fail to deliver quality instruction in an online setting. Instructors also face challenges in the transition process. Therefore, higher institutions must provide instructors with comprehensive training (technical tools, resources, psychological support). When the instructors can provide quality online courses and sufficient support to students, the students’ satisfaction level with online learning will improve. Regarding assignments, instructors have to control the workload of assignments. Some simple and unnecessary appointments should be appropriately deleted. Besides, students need instant feedback and guidance from their instructors. Teachers should respond to students’ projects and give personalized advice. Regarding internet self-efficacy, it may be helpful for higher education institutions to provide appropriate training in internet skills and online course system operation to improve students’ internet self-efficacy.

## 6. Conclusion

It can be concluded from the results that students were not, overall, satisfied with online learning during the pandemic. Additionally, it can be seen that assignments combined with internet self-efficacy will lead to satisfaction with online learning. Furthermore, the combination of the instructor with assignments was the core factor. Internet self-efficacy is also indispensable to explaining higher levels of satisfaction with online learning. However, other factors that were the most influential factors on student satisfaction in previous research, such as interaction and faculty service, were not important in this research.

Although universities were forced to switch to online education during the COVID-19 pandemic, the pandemic has accelerated the development of online teaching. However, universities and teachers may not be fully prepared for this transition. Students who have adapted to the traditional teaching mode inevitably have difficulty in adapting to online courses. Therefore, it is necessary to study students’ satisfaction and the factors influencing it. This study contributes to our understanding of university students’ satisfaction with online learning during the COVID-19 pandemic. A limitation of this study is that we did not consider the influence of students’ major and their levels. Students from majors requiring practical operation and experiments might be less satisfied with online courses. Another limitation is that we did not explore why interaction was not important to students’ satisfaction in this study. A further study could compare the satisfaction of different major students from different levels and could conduct interviews with participants to further verify the influencing factors.

## Data availability statement

The raw data supporting the conclusions of this article will be made available by the authors, without undue reservation.

## Ethics statement

Ethical review and approval was not required for the study on human participants in accordance with the local legislation and institutional requirements. The patients/participants provided their written informed consent to participate in this study.

## Author contributions

The author confirms being the sole contributor of this work and has approved it for publication.

## Conflict of interest

The author declares that the research was conducted in the absence of any commercial or financial relationships that could be construed as a potential conflict of interest.

## Publisher’s note

All claims expressed in this article are solely those of the authors and do not necessarily represent those of their affiliated organizations, or those of the publisher, the editors and the reviewers. Any product that may be evaluated in this article, or claim that may be made by its manufacturer, is not guaranteed or endorsed by the publisher.
